# Accumulation of G_D1α_ Ganglioside in MDA-MB-231 Breast Cancer Cells Expressing ST6GalNAc V

**DOI:** 10.3390/molecules20046913

**Published:** 2015-04-16

**Authors:** Sandy Vandermeersch, Jorick Vanbeselaere, Clément P. Delannoy, Aurore Drolez, Caroline Mysiorek, Yann Guérardel, Philippe Delannoy, Sylvain Julien

**Affiliations:** 1 Structural and Functional Glycobiology Unit, UMR CNRS 8576, University of Lille, 59655 Villeneuve d’Ascq, France; E-Mails: sandy.vandermeersch@gmail.com (S.V.); jorick.vanbeselaere@gmail.com (J.V.); cl-ment.delannoy@etudiant.univ-lille1.fr (C.P.D.); yann.guerardel@univ-lille1.fr (Y.G.); sylvain.julien@univ-lille1.fr (S.J.); 2 Blood Brain Barrier Laboratory (LBHE), EA 2465, University of Artois, 62300 Lens, France; E-Mails: aurore.drolez@univ-artois.fr (A.D.); cj.mysiorek@gmail.com (C.M.); 3 Cell Plasticity and Cancer, INSERM U908, University of Lille, 59655 Villeneuve d’Ascq, France

**Keywords:** ST6GalNAc V, breast cancer, MDA-MB-231, α-gangliosides, GD1α

## Abstract

α-Series gangliosides define a particular sub-class of glycosphingolipids containing sialic acid α2,6-linked to GalNAc residue that was isolated as a minor compound from the brain. The sialyltransferase ST6GalNAc V was cloned from mouse brain and showed α2,6-sialyltransferase activity almost exclusively for G_M1b_, to form G_D1α_ and is considered as the main enzyme involved in the biosynthesis of α-series gangliosides. Recently, *ST6GALNAC5* was identified as one of the genes over-expressed in breast cancer cell populations selected for their ability to produce brain metastasis. However, the capacity of human breast cancer cells to produce α-series gangliosides has never been clearly demonstrated. Here, we show by stable transfection and MS-MS analysis of total glycosphingolipids that *ST6GALNAC5* expressing MDA-MB-231 breast cancer cells accumulate G_D1α_ ganglioside (IV^3^Neu5Ac_1_, III^6^Neu5Ac_1_Gg_4_-Cer).

## 1. Introduction

Gangliosides, the glycosphingolipids (GSL) carrying one or several sialic acid residues, are essentially located on the outer leaflet of the plasma membrane where they form lipid rafts with cholesterol and other sphingolipids. Gangliosides were demonstrated to be essential molecules in the modulation of signal transduction pathways by their interactions with signal transduction molecules including receptors tyrosine kinases. Gangliosides are therefore involved in cell adhesion, proliferation and recognition processes [1]. GSL from the ganglio-series are usually classified in four series (0-, a-, b- and c-series) according to the presence of 0 to 3 sialic acid residues linked to lactosylceramide [2]. Normal human tissues mainly express gangliosides from 0- and a-series whereas more ‘complex’ gangliosides from b- and c-series are mainly restricted to the nervous system [3]. The expression of complex gangliosides increases under several pathological conditions including neurodegenerative disorders [4], immune diseases [5] and cancers [6]. For example, G_D3_ and G_D2_ are over-expressed in neuroectoderm-derived tumors such as melanoma, neuroblastoma and triple-negative breast cancer, in which they mediate cell proliferation, migration, tumor growth and angiogenesis [6].

α-Series gangliosides define a particular sub-class of GSL containing Neu5Ac α2,6-linked to the GalNAc residue of the gangliopentaosyl backbone Neu5Acα2-3Galβ1-3GalNAcβ1-4Galβ1-4Glc (IV^3^Neu5Ac_1_G_g4_). The typical α-series ganglioside G_D1α_ (IV^3^Neu5Ac_1_,III^6^Neu5Ac_1_Gg_4_-Cer) was first isolated as a minor compound from rat ascites hepatoma AH 7974F cells [7] and from bovine brain [8], with an expression restricted to particular cell populations of the forebrain, the midbrain and the cerebellum [9]. Three members of the CMP-Neu5Ac: β-N-acetylgalactosaminide α2,6-sialyltransferase family (ST6GalNAc III, V and VI) were shown to catalyze *in vitro* the transfer of a sialic acid residue onto G_M1b_ (IV^3^Neu5Ac_1_Gg_4_-Cer) to form G_D1α_[10]. However, according to its substrate specificity and expression pattern, ST6GalNAc V is generally considered as the main G_D1α_ synthase. ST6GalNAc V cDNA was cloned from mouse brain [11,12] and *st6galnac5* gene is specifically expressed in brain tissues, mostly in forebrain and cerebellum [12]. When expressed as a soluble recombinant protein, the mouse ST6GalNAc V showed α2,6-sialyltransferase activity almost exclusively for G_M1b_, while being inactive toward glycoproteins [11]. The recombinant mouse ST6GalNAc VI was also shown to convert *in vitro* G_M1b_, G_D1a_, and G_T1b_ into α-series gangliosides G_D1α_, G_T1aα_, and G_Q1bα_, respectively [13]. However, this enzyme was lately demonstrated to be responsible for the synthesis of disialyl-Le^a^ but not for α-series gangliosides in human colon tissues [14]. To our knowledge, the enzymatic activity of human ST6GalNAc V was never thoroughly investigated. However, it was shown that transfection of Human ST6GalNAc V into U373MG glioma cells produced the unusual α2,6-monosialoganglioside, G_M2α_ (Neu5Acα2-6GalNAcβ1-4Galβ1-4Glc-Cer, III^6^Neu5Ac_1_G_g3_-Cer) instead of G_D1α_[15].

To date, little is known concerning the specific function of α-series gangliosides. It has been proposed that G_D1α_ could play a role in Purkinje cell functions in the cerebellum [9] and that G_D1α_ could serve as an adhesion molecule for high-metastatic murine lymphosarcoma cell line RAW117-H10 in the adhesion to hepatic sinusoidal endothelial cells [16]. *ST6GALNAC5* gene was also shown playing a role in HeLa cell adhesion [17,18] and recently, *ST6GALNAC5* was identified as one of the genes over-expressed in breast cancer cell populations selected for their ability to produce brain metastasis [19]. ShRNA inhibition of *ST6GALNAC5* expression reduced the capacity of breast cancer cells to produce brain metastasis whereas the expression of *ST6GALNAC5* in parental cell lines promoted brain metastasis formation [19]. Moreover, *ST6GALNAC5* was demonstrated as the only gene specifically correlated with brain metastasis of breast cancer and up-regulated in human brain metastasis samples [20]. However, the capacity of Human breast cancer cells that express *ST6GALNAC5* to produce α-series gangliosides has never been clearly demonstrated. Here, we show by MS analysis of total GSL that *ST6GALNAC5* expressing MDA-MB-231 breast cancer cells accumulate G_D1α_ ganglioside.

## 2. Results and Discussion

### 2.1. Quantitative Real-Time-PCR (qPCR) Analysis of ST6GalNAc V Expression in Transfected MDA-MB-231 Cells

**Figure 1:**
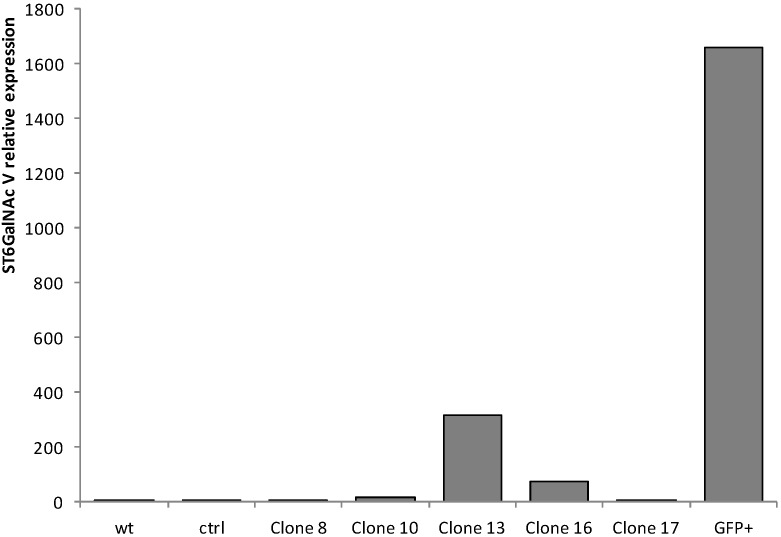
QPCR analysis of ST6GalNAc V expression in control and transfected MDA-MB-231 cells. Quantification of ST6GalNAc V expression was performed by the method described by Pfaffl [21] and normalized to HPRT. The expression of ST6GalNAc V was relative to wild-type (wt), which was regarded as 1. Ctrl, control cells transfected with the empty vector; GFP+, GFP-positive cell population.

**Figure 2:**
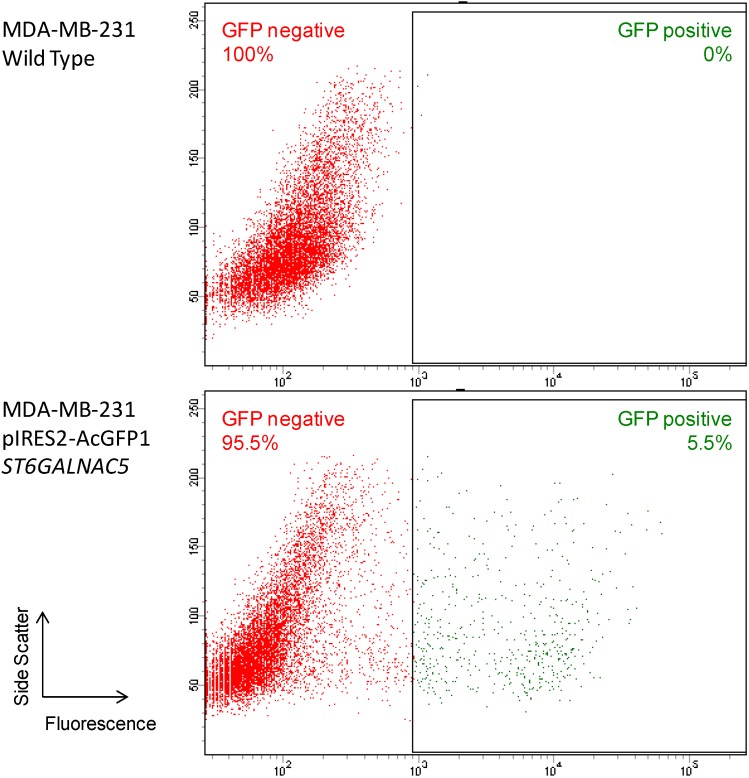
Cell sorting for GFP expression of the G418-resistant cell population. The G418-resistant cell population was sorted for GFP expression on an ARIA SORP flow cytometer.

MDA-MB-231 cells were transfected with the pIRES2-AcGFP1 expression vector containing the full-length cDNA of human ST6GalNAc V or the empty vector as control. pIRES2-AcGFP1 is a bicistronic expression vector designed for the simultaneous expression of green fluorescent protein (GFP) and a protein of interest in mammalian cells. Transfected cells were cultured 14 days in the presence of 1 mg/mL G418. Eighteen individual G418-resistant colonies were isolated by limiting dilution and analyzed for the expression of ST6GalNAc V transcripts. No amplification was obtained for thirteen of the resistant clones (not shown) and of the 5 clones showing over-expression of ST6GalNAc V mRNA compared to control cells, the clone #13 displayed the highest level (317-fold) (Figure 1). In parallel, the polyclonal G418-resistant cell population was sorted for GFP expression and 5.5% of the cell population was selected (Figure 2). 

The resulting GFP-positive polyclonal cell population was analyzed for the expression of ST6GalNAc V and shown a high level of ST6GalNAc V mRNA expression compared to control cells (1657-fold) (Figure 1).

### 2.2. Flow Cytometry Analysis of α2,6-Sialylation Using Sambucus Nigra Agglutinin (SNA)

**Figure 3:**
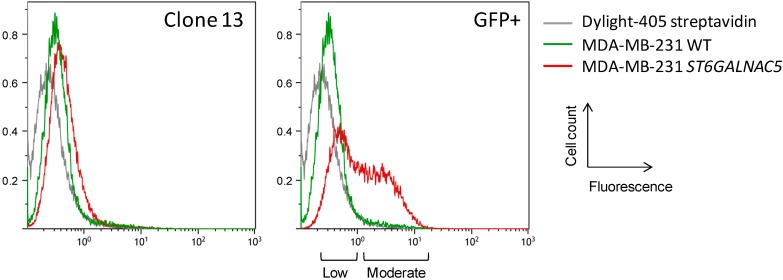
Flow cytometry analysis of α-2,6-sialylation in ST6GalNAc V transfected MDA-MB-231 cells. Detection of α-2,6-sialylation was performed using biotin-labeled SNA and revealed with Dylight-405-conjugated streptavidin. WT, wild-type.

The α-2,6-sialylation of clone #13 and polyclonal GFP-positive cell population was analyzed by flow cytometry using SNA lectin that binds to Neu5Acα2,6-Gal/GalNAc sequence [22]. SNA binding to clone #13 was slightly increased compared to wild-type MDA-MB-231 (Figure 3). In parallel, SNA binding to the polyclonal GFP-positive cell population was stronger but heterogeneous, indicating the presence of at least two populations displaying ‘low’ and ‘moderate’ staining. According to the observed staining, the clone #13 may therefore derive from the low staining sub-population. To our knowledge, the affinity of SNA to Neu5Acα2-3Galβ1-3[Neu5Acα2-6]GalNAcβ- tetrasaccharide has never been clearly determined. Most (if not all) the glycan structures used to analyze SNA binding contain terminal α-2,6-linked sialic acid [23] and the affinity of the lectin could be reduced when sialic acid is α-2,6-linked to an internal GalNAc residue substituted by Neu5Acα2-3Galβ1-3 sequence. This could explain that only a slight increase of SNA binding was observed only for the GFP-positive cell population.

### 2.3. MS Analysis of GSL in ST6GalNAc V Transfected MDA-MB-231 Cells

**Figure 4:**
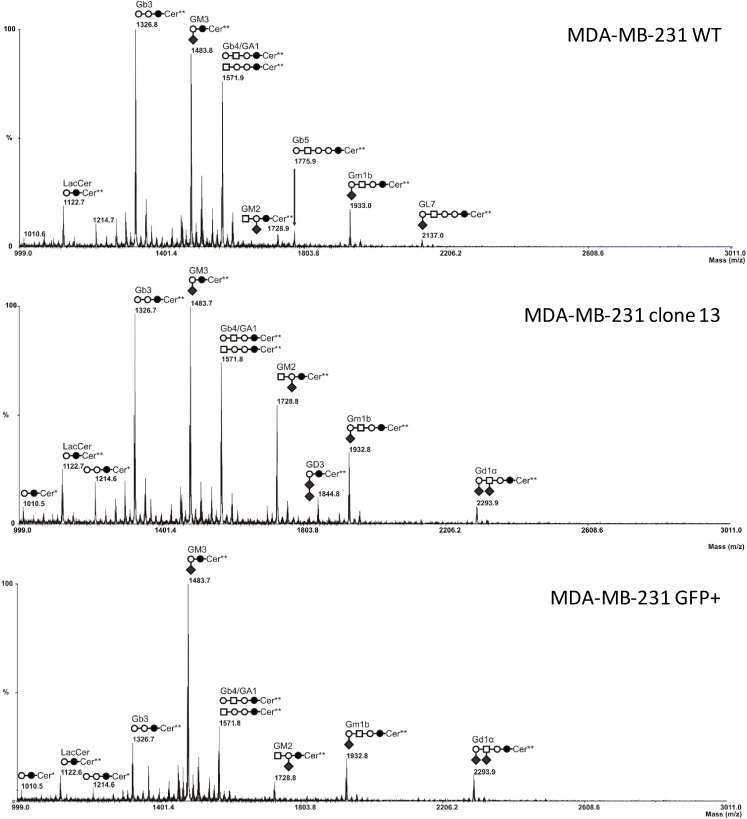
Comparison of MS profiles of permethylated glycosphingolipids purified from MDA-MB-231 wt, clone #13 and GFP+ ST6GalNAc V transfected cells. GSL are present as d18:1/C16:0 (Cer*) and d18:1/C24:0 (Cer**) isomers. ○, Gal; ●, Glc; □, GalNAc; ♦, Neu5Ac.

Total glycosphingolipids were extracted from control and ST6GalNAc V expressing cells, purified by reverse phase chromatography and permethylated prior to MS analysis. As previously shown [24], wild-type or empty vector-transfected MDA-MB-231 (not shown) cells expressed neutral globosides G_b3_ and G_b4_ and monosialylated gangliosides, mainly G_M3_ (Figure 4A). The precursor lactosylceramide was also detected, as well as a monosialoganglioside at *m*/*z* 1933, which was confirmed to correspond to G_M1b_ by MALDI-TOF/TOF fragmentation analysis (data not shown). Two ceramide isoforms are commonly expressed in human tissues due to the substitution of the sphingosine moiety by palmitic acid C16:0 (Cer*) or lignoceric acid C24:0 (Cer**) (Figure 4). 

As shown in Figure 4B,C, the composition in GSL of clone #13 and polyclonal GFP-positive cell population was similar to control cells with the notable expression at an additional signal at *m*/*z* 2294.5 that was tentatively identified as an isomer of G_D1_ ganglioside (G_D1a_, G_D1b_, G_D1c_ or G_D1__α_) with 3 hexoses, one N-acetylhexosamine and 2 N-acetylneuraminic acid residues. Surprisingly, the presence of G_D3_ at *m*/*z* 1,844.8 was also noticed in clone #13. However G_D3_ was not detected in the polyclonal GFP+ cell population, despite its higher level of *ST6GALNAC5* transcripts (Figure 2). This suggests that G_D3_ expression in clone #13 is probably an artefact due to the clone selection process rather than a consequence of *ST6GALNAC5* expression.

**Figure 5:**
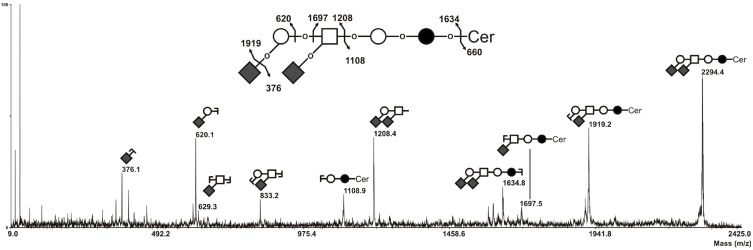
MS/MS sequencing of permethylated G_D1_ at *m*/*z* 2295 with ceramide moieties d18:1/C24:0 (Cer**). All fragments are observed as [M+Na]^+^ adducts. Fragment ions were annotated according to nomenclature of Domon and Costello [26]. The nature of monosaccharides was deduced from known biosynthesis of gangliosides. ○, Gal; ●, Glc; □, GalNAc; ♦, Neu5Ac.

MALDI-TOF/TOF fragmentation analysis established that this signal corresponded to G_D1__α__,_ as shown in Figure 5. Indeed, the [M+Na]^+^ B/Y-ions at *m*/*z* 1208/1108 attested the presence of a terminal HexNAc_1_Hex_1_Neu5Ac_2_ tetrasaccharide, excluding G_D1a_ and G_D1b_ isomers in which at least one N-acetylneuraminic acid residue is linked to the internal galactose residue and characterised by B/Y-ions at *m*/*z* 847/1,469 and *m*/*z* 486/1,830, respectively [25]. Then, the presence of [M+Na]^+^ secondary ion at *m*/*z* 629 testified the presence of an internal HexNAc_1_Neu5Ac_1_ disaccharide unit, characteristic from the α-series gangliosides. Finally, the absence of [M+Na]^+^ B-ions at *m*/*z* 759, which corresponds to a disialylated sequence finally excluded G_D1c_ isomer. Altogether, these data clearly demonstrate that the additional signal at *m*/*z* 2,295 that appeared in the GSL composition of clone #13 and GFP-positive cell population corresponded exclusively to G_D1__α_. 

## 3. Experimental Section

### 3.1. Cell Culture and Transfection

The breast cancer cell line MDA-MB-231 was obtained from the American Type Cell Culture Collection (Manassas, VA, USA) Cell culture reagents were purchased from Lonza (Levallois-Perret, France). Cells were routinely grown in monolayer and maintained at 37 °C in an atmosphere of 5% CO_2_, in Dulbecco’s modified Eagle’s medium (DMEM) supplemented with 10% fetal bovine serum (FBS), 2 mM l-glutamine, and 100 units/mL penicillin-streptomycin. The full-length human ST6GalNAc V cDNA [11] was amplified by PCR from the Mammalian Gene Collection (MGC) clone 3356535 using sense 5'-gtagctagctcgagatgaagaccctgatgcgccatgg-3' and antisense 5'-atatatagatctgaattctcagtgtctcggtgtctgatgc-3' primers containing *EcoR*I and *Xho*I restriction sites, respectively (underlined) and inserted into the *EcoR*I and *Xho*I sites of the bicistronic pIRES2-AcGFP1 expression vector designed for the simultaneous expression in mammalian cells of green fluorescent protein (GFP) and the protein of interest (Clontech, Mountain View, CA, USA). The resulting plasmid was purified using NucleoSpin purification kit (Macherey-Nagel, Hoerdt, France) according to manufacturer’s instructions and fully sequenced. Transfection was performed by lipofection using Lipofectamine^®^ 2000 (Invitrogen, Carlsbad, CA, USA). After transfection, cells were maintained for 48 h in DMEM at 37 °C in an atmosphere of 5% CO_2_ and then cultured in the presence of 1 mg/mL G418 (Invitrogen). After 14 days in the selective medium, individual G418-resistant colonies were isolated by limit dilution. Alternatively, the G418-resistant cell population was sorted for GFP expression on an ARIA SORP flow cytometer (BD Biosciences, Le Pont de Claix, France). Control cells (empty vector transfected) and ST6GalNAc V positive cells were cultured in the presence of 1 mg/mL G418 (Invitrogen).

### 3.2. QPCR Analysis of ST6GalNAc V

Total RNA was extracted using the Nucleospin RNA II kit (Macherey Nagel), quantified using DS-11 spectrophotometer (Denovix, Wilmington, DE, USA) and the purity of the preparation was checked by ratio of the absorbance at 260 and 280 nm. The cDNA was synthesized with 2 µg of RNA using the Maxima first strand cDNA Synthesis kit (Thermo Fisher Scientific, Langenselbold, Germany). PCR primers for Hypoxanthine PhosphoRibosylTransferase (HPRT) were previously described [27]. Primers for ST6GalNAc V (sense: 5'-ggatcccaatcacccttcag-3', antisense: 5'-tagcaagtgattctggtttcca-3') were designed using Primer 3 software. QPCR reactions (25 µL) were performed using Maxima SYBR Green Fluorescein qPCR MasterMix (Thermo Fisher Scientific), with 2 µL of cDNA solution and 300 nM final concentration of each primer in a Mx3005p qPCR System (Stratagene, La Jolla, CA, USA). PCR conditions were: 95 °C for 30 s, 51 °C for 45 s, 72 °C for 30 s (40 cycles). Assays were performed in triplicate and ST6GalNAc V transcript expression level was normalized to HPRT using the method described by Pfaffl [21]. Serial dilutions of the appropriate positive control cDNA sample were used to create standard curves for relative quantification and negative control reactions were performed by replacing cDNA templates by sterile water.

### 3.3. Flow Cytometry Analysis

Cells (3 × 10^5^) were washed in cold PBS and detached by: 5 mM ethylenediaminetetraacetic acid (EDTA). Cells were incubated at 4 °C during 1 h with 10 µg/mL Biotin-labeled SNA (Vector Laboratories, Burlingame, CA, USA) diluted in phosphate buffered saline (PBS) containing 1% bovine serum albumin (PBS-BSA) (Sigma-Aldrich, St. Louis, MO, USA). After washing with PBS-BSA, cells were incubated 30 min on ice with Dylight-405-conjugated streptavidin (Jackson Immunoresearch, West Grove, PA, USA) and analyzed by flow cytometry (Cyan ADP Analyzer, Beckman Coulter, Lille, France). 

### 3.4. Extraction and Preparation of Glycolipids

Twenty dishes (10 cm diameter) of cultured cells were washed twice with ice-cold PBS and cells were sonicated on ice in 200 µL of water. The resulting material was dried under vacuum and sequentially extracted by CHCl_3_/CH_3_OH (2:1, v/v), CHCl_3_/CH_3_OH (1:1, v/v) and CHCl_3_/CH_3_OH/H_2_O (1:2:0.8, v/v/v). Supernatants were pooled, dried and subjected to a mild saponification in 0.1 M NaOH in CHCl_3_/CH_3_OH (1:1) at 37 °C for 2 h and then evaporated to dryness [28]. Samples were reconstituted in CH_3_OH/H_2_O (1:1, v/v) and applied to a reverse phase C_18_ cartridge (Waters, Milford, MA, USA) equilibrated in the same solvent. After washing with CH_3_OH/H_2_O (1:1, v/v), GSL were eluted by CH_3_OH, CHCl_3_/CH_3_OH (1:1, v/v) and CHCl_3_/CH_3_OH (2:1, v/v).

### 3.5. Mass Spectrometry Analysis of GSL

Prior to mass spectrometry analysis, GSL were permethylated according to Ciucanu and Kerek [29]. Briefly, compounds were incubated 2 h in a suspension of 200 mg/mL NaOH in dry DMSO (400 µL) and CH_3_I (200 µL). The methylated derivatives were extracted in CHCl_3_ and washed several times with water. The reagents were evaporated and the sample was dissolved in CHCl_3_ in the appropriate dilution. MALDI-MS and MS/MS analyses of permethylated GSL were performed on 4800 Proteomics Analyzer (Applied Biosystems, Framingham, MA, USA) mass spectrometer, operated in the positive reflectron mode. For MS acquisition, 5 µL of diluted permethylated samples in CHCl_3_ were mixed with 5 µL of 2,5-dihydroxybenzoic acid matrix solution (10 mg/mL dissolved in CHCl_3_/CH_3_OH (1:1, v/v)). The mixtures (2 µL) were then spotted on the target plate and air dried. MS survey data comprises a total of 50 sub-spectra of 1500 laser shots. Peaks observed in the MS spectra were selected for further MS/MS. CID MS/MS data comprises a total of 100 sub-spectra of 3000 laser shots. Two or more spectra can be combined post-acquisition with mass tolerance set at 0.1 Da to improve S/N ratio. The potential difference between the source acceleration voltage and the collision cell was set to 1 kV and argon was used as collision gas.

## 4. Conclusions

The α-series gangliosides define a rare subclass of GSL essentially restricted to some area of mammalian brain. α-gangliosides biological function, however, remains mostly unknown. Based on the substrate specificity of soluble recombinant enzymes, the α2,6-sialytransferase ST6GalNAc V is considered as the main G_D1α_ synthase. Strikingly, ST6GalNAc V expression is also restricted to the brain. The identification of *ST6GALNAC5* as one of the genes involved in breast cancer brain metastasis [19] raised the question of the capacity of breast cancer cells to synthesize α-series gangliosides. Here, we show for the first time that the expression of human ST6GalNAc V cDNA in human cancer cells (MDA-MB-231) results in the accumulation of G_D1α_. However, the question of the role of α-series gangliosides in breast cancer brain metastasis remains open. To our knowledge, no recognition protein was identified to date to specifically bind α-series gangliosides [30]. *ST6GALNAC5* gene was previously identified playing a role in controlling the degree of cell adhesion in Hela cells. It was shown that higher *ST6GALNAC5* transcription correlated with a lower degree of adhesion, siRNA inhibition of *ST6GALNAC5* transcription being followed by enhanced adhesion [18]. Furthermore, the expression of *ST6GALNAC5*, presumably increasing the expression of α-series gangliosides in breast cancer cells could promote their capacity to form brain metastasis. Although the authors showed *ST6GALNAC5* over-expression increased transmigration through a brain-like endothelial barrier, the *in vitro* model using HUVEC endothelial cells may be questionable. Further investigation are required in order to delineate the molecular mechanism that allows the recognition of α-series gangliosides and the role of these glycolipids in the brain metastasis cascade.

## Abbreviations

BAS: Bovine Serum Albumin
Cer: ceramide
DMEM: Dulbecco’s Modified Eagle’s Medium
FBS: Fetal Bovine Serum
GFP: green fluorescent protein
GSL: glycosphingolipid
HPRT: Hypoxanthine PhosphoRibosylTransferase
LacCer: Lactosylceramide
MALDI-TOF: matrix assisted laser desorption-ionization time-of-flight
MS: Mass Spectrometry
PBS: Phosphate Buffered Saline
PCR: Polymerase Chain Reaction
qPCR: Quantitative real-time PCR
SNA: *Sambucus nigra* agglutinin
WT: Wild-Type
